# Rethinking the prognosis model of differentiated thyroid carcinoma

**DOI:** 10.3389/fendo.2024.1419125

**Published:** 2024-09-30

**Authors:** Liang He, Jingzhe Xiang, Hao Zhang

**Affiliations:** Department of Thyroid Surgery, The First Hospital of China Medical University, Shenyang, China

**Keywords:** differentiated thyroid carcinoma, cancer-specific survival, decision tree methodology, proportions of variance explained, TNM staging system

## Abstract

**Background:**

The prediction efficiency of long-term cancer-specific survival (CSS) in guiding the treatment of differentiated thyroid carcinoma (DTC) patients is still unsatisfactory. We need to refine the system so that it more accurately correlates with survival.

**Methods:**

This is a retrospective study using the Surveillance, Epidemiology, and End Results (SEER) database, and included patients who underwent surgical treatment and were diagnosed with DTC from 2004 to 2020. Patients were divided into a training cohort (2004–2015) and validation cohort (2016–2020). Decision tree methodology was used to build the model in the training cohort. The newly identified groups were verified in the validation cohort.

**Results:**

DTC patient totals of 52,917 and 48,896 were included in the training and validation cohorts, respectively. Decision tree classification of DTC patients consisted of five categorical variables, which in order of importance were as follows: M categories, age, extrathyroidal extension, tumor size, and N categories. Then, we identified five TNM groups with similar within-group CSS. More patients were classified as stage I, and the number of stage IV patients decreased significantly. The new system had a higher proportion of variance explained (PVE) (5.04%) and lower Akaike information criterion (AIC) (18,331.906) than the 8th TNM staging system (a PVE of 4.11% and AIC of 18,692.282). In the validation cohort, the new system also showed better discrimination for survival.

**Conclusion:**

The new system for DTC appeared to be more accurate in distinguishing stages according to the risk of mortality and provided more accurate risk stratifications and potential treatment selections.

## Introduction

As the most common type of thyroid cancer, the incidence of differentiated thyroid carcinoma (DTC) has shown a sharp rise over the past 30 years (1). To define treatment and evaluate the prognosis of patients, the American Joint Committee on Cancer (AJCC) released a new tumor-node-metastasis (TNM) system, which was based on long-term follow-up surveillance and survival diversities from population studies (2, 3). The American Thyroid Association (ATA) (4) and the British Thyroid Association (BTA) (5) regarded it as a guideline for classifying patients at the initial presentation, determining the cancer-specific survival and best initial treatment. In fact, the actual goal of DTC management is to predict recurrence, but we should also pay attention to its long-term CSS.

Age is considered as the most important prognostic factor and has been combined with the anatomic tumor extent to stage DTC since 1983 (6, 7). Although many studies (8–10) have indicated that distant metastasis has the highest hazard ratio of DTC, the TNM system still adopted age as the most important and initial dichotomous variable in the postoperative staging system over the last 40 years, rather than distant metastasis. The division by age resulted in a poor correlation between the risk of death and stage: patients with stage II disease could have a low, intermediate, or even high risk of death (11).

As knowledge of cancer biology evolves, diagnostic tools and treatment modalities have been improved constantly (3). Although the TNM staging system has been widely applied in clinic, there are still studies that query the predictive effectiveness on an individual level (10, 12–15). For example, for a 54-year-old patient with T4bN1bM1, the TNM stage should be set as stage II, and for a 56-year-old patient with T1aN1aM0, the TNM stage is also stage II. If the postoperative stage of the patient is T4aN0M0, it can even be set as stage III. This is obviously unreasonable. The balance between age and distant metastasis is controversial. A model combined with multiple factors should be built.

Recently, decision tree methodology has become popular in medical research (16–18). An example of the medical use of the decision tree is to diagnose a disease according to the symptoms, in which the categories defined by the decision tree can be different clinical subtypes or a disease, or the prognosis of different patients (19). Therefore, we aimed to use a statistically sophisticated methodology to identify cancer-specific survival (CSS) in individual patients with DTC to define more accurate staging groups than previous versions.

## Materials and methods

### Data source

Data were extracted from the Surveillance, Epidemiology, and End Results (SEER) database due to its large sample size. The SEER Program of the National Cancer Institute (20) is a dependable national cancer registry that is widely used within the USA. SEER currently collects and publishes cancer incidence and survival data from population-based cancer registries covering an estimated 26% of the US population. The mortality rate data reported by SEER are provided by the National Cancer Institute, updated annually, and provided as a public service in print and electronic formats.

### Patients

All included patients had undergone initial surgery and been diagnosed with papillary thyroid carcinoma (PTC) or follicular thyroid carcinoma (FTC) by postoperative pathology between 2004 and 2020. They were identified using the histopathology codes of the International Classification of Disease for Oncology, third edition (ICD-O-3): 8050/3, 8260/3, 8340/3, 8341/3, 8342/3, 8343/3, 8344/3, 8330/3, 8331/3, 8332/3, and 8335/3. The exclusion criteria were as follows: (1) unknown age or race; (2) unclear/missing surgical information; (3) incomplete/missing information regarding tumor size, tumor extension, lymph node metastasis, or distant metastasis; and (4) the SEER cause-specific death classification was “N/A not first tumor”. Because there are very few cases of distant metastasis in our center, we did not use the information from our center. All patients with missing data were excluded. Patients were divided into a training cohort (2004-2015) and a validation cohort (2016-2020). Given that the SEER database provides anonymous and public access to its data after obtaining permission, the Institutional Review Committee was not required to scrutinize this study.

General patient information was extracted from the database, including age at diagnosis, gender, histological type, and tumor size. It is worth noting that our data were somewhat different from those previously. Tumor size was divided into three subgroups (≤1 cm, 1–4 cm, and >4cm and extrathyroidal extension (ETE) had four subgroups (no, only strap muscles, T4a and T4b). CSS was defined as the time from initial surgery to the last censoring or death caused by DTC (21, 22).

### Statistical analysis

All categorical variables were reported as frequency and proportion. CSS was estimated using the Kaplan–Meier method. The effect of potential predictors was estimated using Cox regression, and results were reported as a hazard ratio (HR) with a 95% confidence interval (CI). The validity of the model was examined with the area under the receiver operating characteristic curve (AUC), proportions of variance explained (PVEs), and the Akaike information criterion (AIC). AUC was displayed in the output window. The value of the AUC ranges from 0 to 1, with values closer to 1 indicating a stronger discriminative ability of the model. A mathematical formula of PVE = 1 - exp(-G²/n) was used to determine PVE, where ‘G²’ was the maximum likelihood ratio determined by the chi-square test and Cox regression analyses, and ‘n’ was the total number of cases in the present study. PVEs (%) range from 0 to 100; larger numbers suggest a better predictability (23, 24). The AIC was defined as follows: AIC =-2 log maximum likelihood + 2 *(the number of parameters in the model). A smaller AIC value indicated a better goodness-of-fit (25).

### Decision tree

Decision tree methodology is a commonly used data mining method that is used in many applications as a powerful solution to classification and prediction problems. The method divides a data group into branch segments, and constructs an inverted tree model including root nodes, internal nodes, and leaf nodes. The Gini index is used to measure the change in “purity” of a variable after node splitting. When merging variables, the model seeks a splitting method that minimizes the Gini index. If two or more variables produce similar purity after splitting, they may be merged. It is a fast and effective method and can provide good decision support (26). The goal of a classification tree is to separate the observations belonging to one category from those belonging to another category as far as possible through a series of binary data segmentations (27).

Several algorithms have been introduced to construct decision trees, such as classification and regression trees (CARTs). The CART algorithm is one of the most commonly used methods of constructing decision trees. It was applied to our training cohort to divide the patients into several groups. We then combined adjacent groups based on their statistical properties and clinical experience to obtain the new staging system. Contrasts between adjacent stage groups were evaluated by Cox regression analysis, adjusting for baseline factors. The methodology is a built-in computational feature in SPSS, requiring no additional algorithms.

### Validation cohort

The new TNM groupings and the 8^th^ AJCC TNM staging system were applied to the validation cohort to examine CSS estimates. The estimates obtained using the new TNM groupings were compared with those obtained using the 8^th^ AJCC TNM staging system. All statistical calculations were performed using IBM SPSS software (version 23.0) and R, version 3.1.0 (R Project).

## Results

### Training cohort

A total of 52,917 patients with DTC were included in the training cohort. Demographic and clinical patient characteristics are shown in **Table 1**. Of these, 40,848 (77.2%) were women. In the pre analysis, we firstly included age as a continuous variable, and we found that the cohort was divided into 17 subgroups, which made the system unsuitable for clinical work. Additionally, it was found that the current staging system does not perform better than the 8th AJCC TNM staging system; therefore, we still regarded it as a binary variable (<55 and ≥55). Most of the patients (96.9%) had PTC, and 18,148 (34.3%) had a tumor less than 1 cm in size. Overall, 88.7% of our cohort had no extrathyroidal extension after the initial surgery. The invasion of strap muscles and posterior neck compartments (T4a & T4b) were 7.1% and 4.2%, respectively. The incidence of cervical lymph node metastasis was 39.1% and 1.2% (654 patients) had distant metastasis. The proportions of patients at the 8^th^ edition stages I, II, III, IVA, and IVB were 85.7% (n=45,332), 11.7% (n=6,199), 1.3% (n=704), 0.5% (n=287), and 0.7% (n=395), respectively. The mean follow-up duration until censoring or death was 7.3 years. The proportion of cancer-specific deaths in the training cohort was 1.9% (n=995).

**Table 1:** Baseline clinicopathological characteristics of patients in the training cohort.

	Case no. (%) (n=52,917)
Gender	
Female	40,848 (77.2)
Male	12,069 (22.8)
Age (years)	
<55	36,038 (68.1)
≥55	16,879 (31.9)
Histology	
PTC	51,278 (96.9)
FTC	1,639 (3.1)
Foci	
Solitary	27,866 (52.6)
Multifocal	25,051 (47.4)
Tumor size (cm)	
≤1	18,148 (34.3)
1~4	30,251 (57.2)
>4	4,518 (8.5)
ETE	
no	46,958 (88.7)
Strap muscles	3,749 (7.1)
T4a	1,569 (3.0)
T4b	641 (1.2)
N component	
N0	32,240 (60.9)
N1a	14,064 (26.6)
N1b	6,613 (12.5)
Distant metastases	
N0 M0	52,263 (98.8)
Yes M1	654 (1.2)
TNM-8 stage	
I	45,332 (85.7)
II	6,199 (11.7)
III	704 (1.3)
IVA	287 (0.5)
IVB	395 (0.7)
Extent of surgery	
LTT	3,989 (7.5)
TT	48,928 (92.5)
CSS	
Alive/other causes	51,922 (98.1)
Dead	995 (1.9)
Follow up (years)	7.3

To determine the prognostic factors affecting DTC and the relevant factors suitable for inclusion in decision tree analysis, Cox proportional hazard regression analysis for variables associated with CSS was performed (**eTable 1** in **Supplementary Table 1**). In univariate and multivariate analyses, except for foci (*p*=0.32), all variables were statistically significantly associated with CSS. All variables were risk factors, except for being female (HR=0.76). To confirm that there were enough patients in each leaf node of the next decision tree analysis module, we combined clinical experiences and the statistics index. Therefore, we finally decided to include these six variables in the initial analysis of the decision tree model (age at diagnosis, tumor size, histology type, ETE, and N and M categories), which can be closer to clinical applications rather than statistical applications.

**Figure 1:**
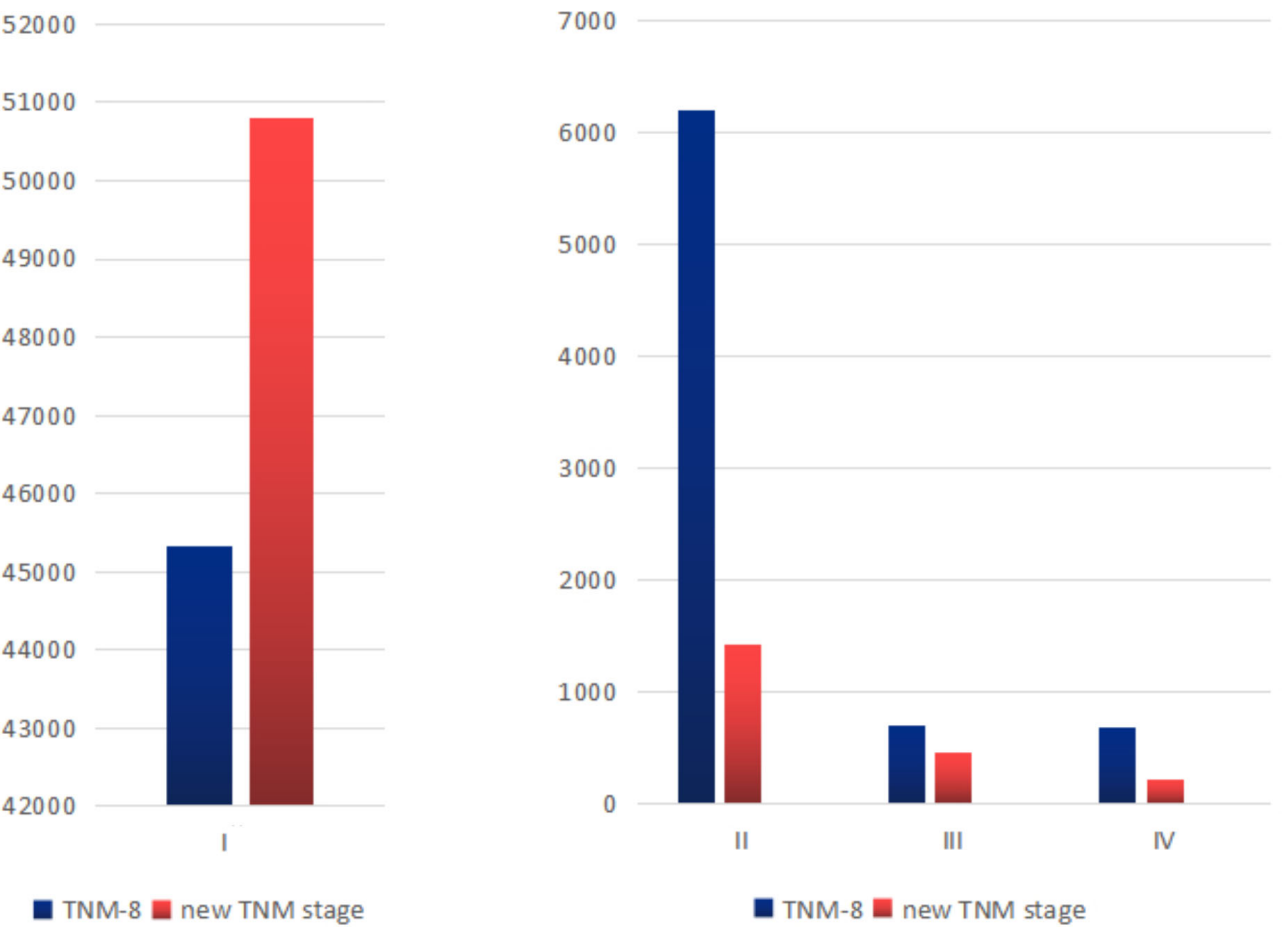
Stage distribution for the TNM-8 stage and new TNM stage in the training cohort.

With the decision tree methodology for the training cohort, M categories had the highest priority, followed by age, ETE, size, and N categories. Histology type had nearly no effect on the decision tree methodology; therefore, it was not shown in the final rules. It is worth noting that (1) in the ETE group, no ETE and only strap muscles were combined into one group and T4a and T4b were combined into one group. We recorded them as T4 later on. (2) In the tumor diameter group, the ≤1cm and 1–4 cm groups were combined into one group and recorded as the ≤4 cm group. Based on the existing interactions between predictor variables, 11 rules were extracted from the decision tree classification corresponding to 11 leaf nodes. These rules are shown in **eTable 2** in the **Supplementary Table 1**. Then, we identified four TNM groups with a similar within-group CSS: stage I was divided into stage IA (M0, age <55, no or only strap muscles, regardless of tumor size and N), stage IB (M0, age ≥55, no or only strap muscles, ≤4 cm or >4 cm with N0; age <55, T4), stage II (M0, age ≥55, no or only strap muscles, >4 cm with N1; M0, age ≥55, T4, ≤4 cm; M1, age <55, T4 stage III (M0, age ≥55, T4, >4 cm; M1, age ≥55, no or only strap muscles, ≤ 4 cm), and stage IV (M1, age ≥55, no or only strap muscles, >4 cm; M1, age ≥55, T4) (**Table 2**). They differed from each other with distinct CSS rates. The differences in the number of patients in each stage are shown in **Figure 1**. More patients were classified as stage I, and the number of stage IV patients decreased.

**Table 2:** New TNM stage derived by decision tree analysis.

Group	M	ETE	Age	Tumor size	N
IA	M0	No or only strap muscles	<55	Any	Any
IB	M0	No or only strap muscles	≥55	≤4 cm	Any
				>4 cm	N0
		T4	<55	Any	Any
II	M0	No or only strap muscles	≥55	>4 cm	N1
		T4		≤4 cm	Any
	M1	Any	<55	Any	Any
III	M0	T4	≥55	>4 cm	Any
	M1	No or only strap muscles		≤4 cm	
IV	M1	No or only strap muscles	≥55	>4 cm	Any
		T4		Any	

**Figure 2:**
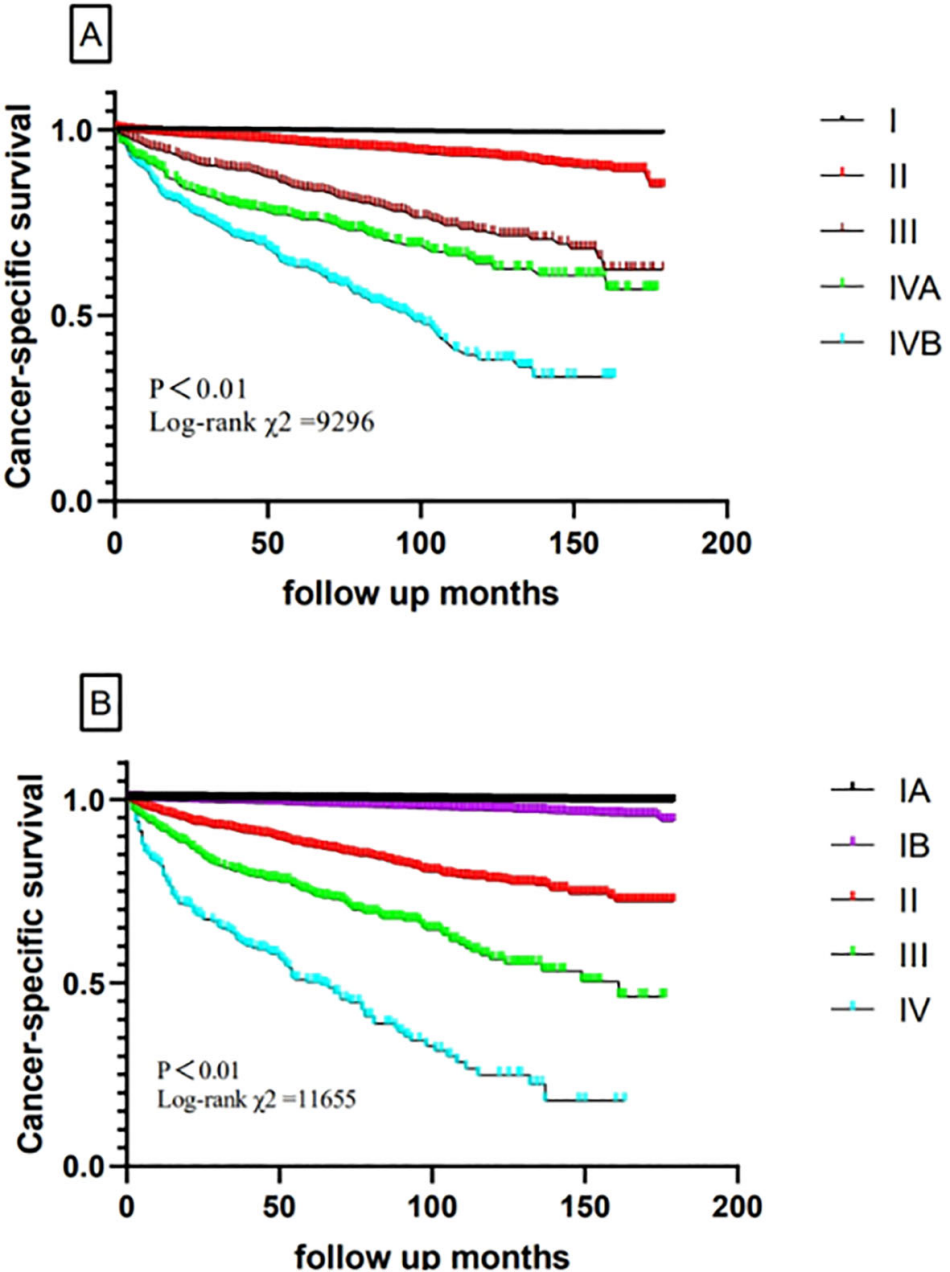
Comparison of cancer-specific survival based on the TNM-8 stage and new TNM stage in the training cohort (**A**, TNM-8; **B**, new TNM stage).

Hazard ratios for the risk of cancer-specific survival and 5-year CSS for the TNM-8 and new TNM stage are displayed in **Table 3** and **Figure 2**. According to our algorithm, differences in survival appeared to be more discriminate in the new TNM groups than the current 8th TNM staging system. In TNM-8, 5-year CSS for DTC was 99.6% for stage I, 96.5% for stage II, 86.0% for stage III, 78.4% for stage IVA, and 66.6% for stage IVB. While in the new system, CSS was 99.8% for stage IA, 98.6% for stage IB, 90.5% for stage II, 77.0% for stage III, and 54.3% for stage IV. The distinction in CSS among different groups has become more significant. The results in **Table 4** demonstrated that the new system could discriminate more accurately than TNM-8. The new system has a higher PVE (5.04%), higher AUC (0.844), and lower AIC (18331.906) than TNM-8 (a PVE of 4.11%, an AUC of 0.819 and an AIC of 18,692.282). Therefore, we assumed that the new staging system revealed a higher efficiency

**Table 3:** Hazard ratios for the risk of cancer-specific survival for the TNM-8 stage and new TNM stage for the SEER cohort.

	No. of patients (%)	No. of deaths (%)	5-year CSS (%)	HR (95% CI)	*P*-value
TNM-8					
I	45,332 (85.7)	270 (0.6)	99.6	Reference	<0.01
II	6,199 (11.7)	329 (5.3)	96.5	9.96 (8.48–11.70)	
III	704 (1.3)	144 (20.5)	86.0	40.72 (33.26–49.85)	
IVA	287 (0.5)	83 (28.9)	78.4	60.90 (47.61–77.89)	
IVB	395 (0.7)	169 (42.8)	66.6	114.73 (94.53–139.24)	
New TNM					
IA	34,788 (65.7)	127 (0.4)	99.8	Reference	<0.01
IB	16,003 (30.2)	357 (2.2)	98.6	6.62 (5.41–8.11)	
II	1,435 (2.7)	242 (16.9)	90.5	53.51 (43.16–66.33)	
III	470 (0.5)	143 (30.4)	77.0	119.81 (94.29–152.25)	
IV	221 (0.4)	126 (57)	54.3	292.84 (228.50–375.30)	

**Table 4:** Comparison between multivariate Cox regression models in the two staging systems.

	Training cohort			Validation cohort		
	AUC	AIC	PVE (%)	AUC	AIC	PVE (%)
TNM-8	0.819	18,692.282	4.11	0.814	5,886.380	3.28
New-TNM	0.844	18,331.906	5.04	0.846	5,706.544	4.00

AUC, area under Roc curve; AIC, Akaike information criterion; PVE, proportions of variance explained.

### Validation cohort

**Figure 3:**
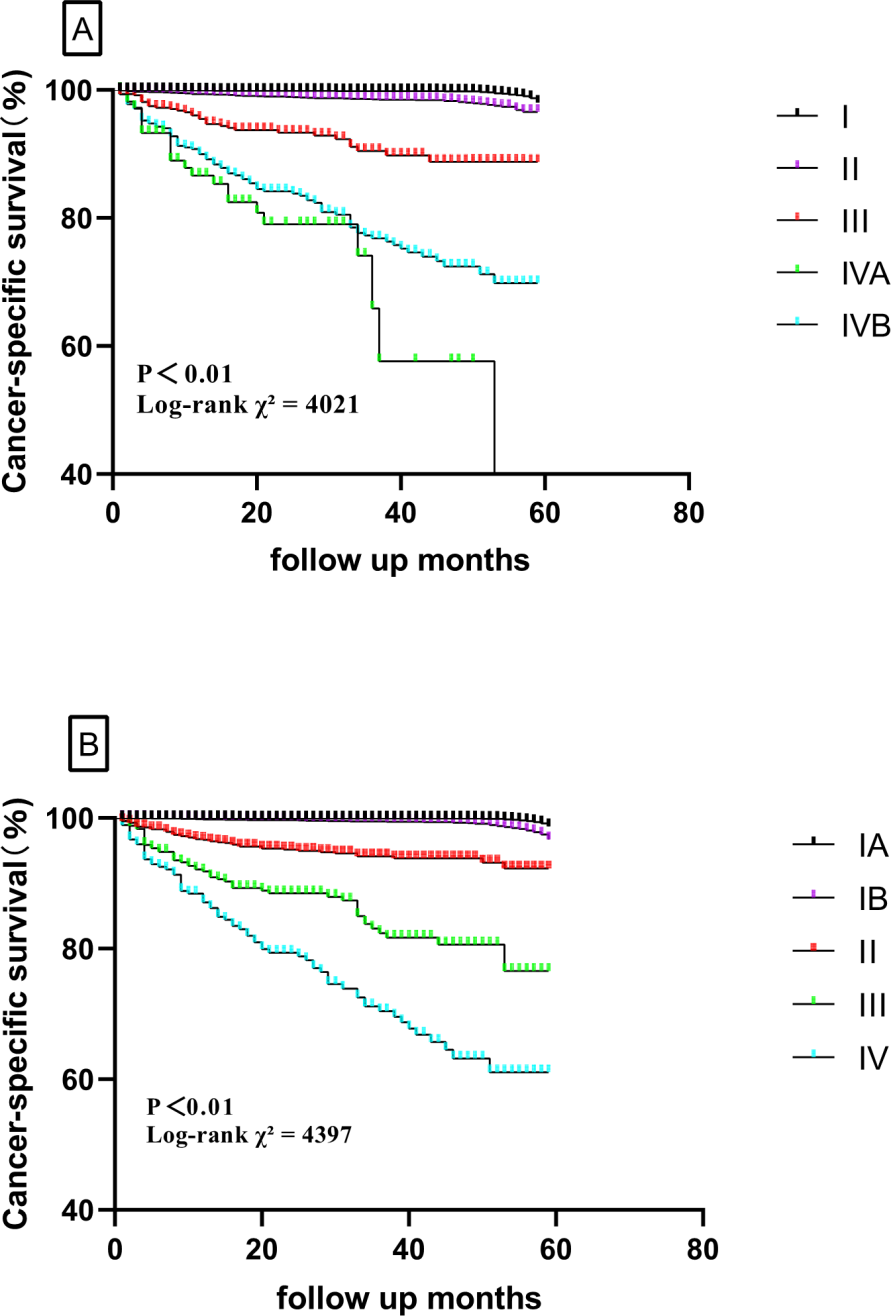
Comparison of cancer-specific survival based on the TNM-8 stage and new TNM stage in the validation cohort (**A**, TNM-8; **B**, new TNM stage).

The validation cohort had a similar population of patients with DTC from SEER (2016–2020). A total of 48,896 patients were finally included in validation analysis. The new TNM system showed a 5-year CSS of 99.9% for stage IA, 99.4% for stage IB, 95.1% for stage II, 84.3% for stage III, and 74.2% for stage IV. For the TNM-8 system, the 5-year CSS was 99.8% for stage I, 98.2% for stage II, 92.6% for stage III, 81.1% for stage IVA, and 80.1% for stage IVB. The 5-year CSS for the two systems is shown in **Figure 3**. The evaluation of discrimination comparison is shown in **Table 4**. These patterns were consistently observed in the training cohort and supported the better discrimination ability of the new system. Accordingly, 5,485 (11.2%) patients were down-staged to stage I. Overall, the new system outperforms the 8th AJCC TNM staging system in all metrics, both in the modeling and validation groups.

## Discussion

The large cohort study examined the appropriateness of the 8th TNM staging system in estimating survival and identifying homogeneous survival groups in DTC patients. It also provided possible revisions to sharpen the estimates of prognosis. Using decision tree methodology, the M category had the highest priority, followed by age, ETE, tumor size, and N categories. Then, we identified four TNM groups (I, II, III, and IV) on the basis of within-group similarities in survival. This approach has been shown to produce accurate predictions. The new groups revealed a higher efficiency than the TNM-8 system, both in the training cohort and validation cohort. After applying the new staging system, nearly two-thirds of patients shifted from stages I-IV to stages IA-III. Based on the SEER cohort, it could better provide accurate predictions and treatment options for DTC patients.

When facing patients with newly diagnosed DTC, the primary task of clinical doctors is to use the available basic prognostic information to make personalized judgments on survival outcomes and make tailored decisions regarding the most effective treatment plan. Therefore, the importance of creating reliable grouping rules to classify patients into some predefined categories is remarkable. Clinical decision rules are designed to help clinicians make diagnosis and treatment decisions (28–31). Although the TNM categories were accepted as descriptors of disease extent, the prediction efficiency of long-term CSS in guiding the clinical treatment of DTC patients was still unsatisfactory.

The decision tree method is a powerful statistical tool for medical research (32, 33). Decision trees have several advantages, such as being able to be applied to any data structure, especially discrete, continuous, or mixed data, and explaining prediction rules in a simple way, providing good accuracy to highly non-linear prediction problems, performing stepwise variable selection, reducing complexity, and so on.

Although the TNM staging system is constantly updated, the 8th edition still has some limitations. Many studies (8–10) have indicated that distant metastasis has the highest hazard ratio for DTC. We indicated that the M category was the most important and initial factor for defining the stages. In addition, in the previous T stage, the diameter of the tumor was not considered for patients with extrathyroidal extension, which was controversial. Previous studies (9, 34, 35) have mentioned that the size of the tumor was also a factor affecting patients in the T3b category, so the tumor diameter should also be considered for these patients. For the two cutoff diameters in our study, 4 cm was accepted by all experts, and we chose 1 cm instead of 2 cm because the topic of active surveillance(AS) (36–38) is controversial at present, and the subtype of papillary thyroid microcarcinoma (PTMC) is removed in the 5th edition of the WHO Classification of Endocrine and Neuroendocrine Tumors (39). Therefore, 1 cm was included in the study as another cutoff point. The results also showed that <1 cm could be combined with 1–4 cm, and there was a survival difference when 4 cm was the cutoff diameter in patients with ETE. Therefore, the tumor diameter was also taken as a factor in the new stage. Furthermore, for the histology type, we did not obtain positive results, which was consistent with the AJCC stage. Nevertheless, many studies (9) have shown that although FTC and PTC are both follicular cell-derived, FTC is more frequent in distant metastasis and predicts a worse prognosis. However, we considered that the difference in histology type disappeared because of the integration of relevant prognostic factors. Finally, the role of lymph node metastasis was further weakened in our staging system and was only slightly reflected in Rules 3 and 4, which were similar to some prognostic scoring systems; the reason for this might be due to the fact that lymph node metastasis (LNM) was more related to recurrence rather than CSS.

The results of the present study demonstrated that our new stage was more suitable for the postoperative staging of DTC. According to the 8th AJCC TNM staging system, 1.2% and 1.3% of patients were classified as having stage IV and III disease; however, according to the new TNM stage, only 0.4% and 0.5% of the patients remained in stage IV and III. This has better benefits in avoiding overtreatment or relieving patients’ emotional stress. We should underline that cancer staging should be based on an accurate correlation with survival, maximizing the similarity of survival within groups and the difference of survival between groups. Although the improvements in AUC, PVE, and AIC are not sufficiently obvious, any system updates should follow the principle of gradual improvement. We proposed a system based on the framework of the TNM-8 system, wishing to provide some support for future amelioration.

The most significant clinical implication of our study is the refinement of the prognostic model for DTC, utilizing a more precise algorithm. We have re-evaluated the risk factors based on their impact on prognosis, resulting in a more rational and efficient system that holds greater significance for the precise and individualized treatment of patients. Furthermore, for DTC, which generally has a favorable prognosis, we also emphasized the importance of patients’ mental health in addition to surgical treatment. The new system reclassifies previously higher-stage patients to lower stages based on our more accurate algorithm, which corresponds to a lighter psychological burden for patients. This multifaceted approach to treatment is an area that we, as surgeons, need to enhance in the future. In clinical settings, we will highlight the potential for improved decision-making regarding follow-up strategies and therapeutic interventions. We will also suggest steps for incorporating this new system into clinical practice, including training for healthcare providers and updates to clinical guidelines to ensure a smooth transition.

### Strengths and limitations

The strengths of this study include its large sample size, the sufficient number of death events, and the application of decision tree methodology. The decision tree model is a non-parametric approach without distributional assumptions that simplifies complex relationships between input variables and is easy to understand and interpret. Any future information about prognostic indicators in DTC could be incorporated with minor changes, making our new staging system adaptable. However, there are also limitations. One potential problem is that the follow-up duration was not long enough; therefore, there might be bias in the results. A longer follow-up period is required in the future. In addition, the outcome is only “alive” or “dead” in the SEER database; biochemical and genetic factors and cancer recurrence were not available. To date, a definition of the genetic events leading to the development of cancer is possible in the vast majority of DTC patients. The translation of biological knowledge into clinical practice represents the next target to be achieved. The prognostication may be empowered in the near future by non-tissue molecular prognosticators, including circulating BRAFV600E, miRNAs, germline VEGF-A SNPs, and so on (40, 41). A great effort is required to overcome the technical issues.

## Conclusion

The new system for DTC appeared to be more accurate in distinguishing stages according to the risk of mortality and provided more accurate risk stratifications and potential treatment selections.

## Data Availability

The raw data supporting the conclusions of this article will be made available by the authors, without undue reservation.
