# Supplementary material for: Rethinking the prognosis model of differentiated thyroid carcinoma
Source: Front Endocrinol (Lausanne). 2024 Sep 30;15:1419125. doi: 10.3389/fendo.2024.1419125 (PMC11471587; doi:10.3389/fendo.2024.1419125)
Supplement: Supplementary file 1 [file Table1.docx]

eTable 1  Clinicopathological parameters associated with cancer-specific survival in training cohort

| Variables | univariate analysis | | | multivariate analysis | | |
| --- | --- | --- | --- | --- | --- | --- |
|  | HR | 95% CI | P-value | HR | 95% CI | P-value |
| Female | 0.35 | 0.31-0.39 | ＜0.01 | 0.76 | 0.67-0.87 | ＜0.01 |
| ≥55 | 9.16 | 7.85-10.69 | ＜0.01 | 7.63 | 6.52-8.94 | ＜0.01 |
| FTC | 2.46 | 1.94-3.12 | ＜0.01 | 1.82 | 1.41-2.35 | ＜0.01 |
| Multifocal | 1.07 | 0.94-1.21 | 0.32 |  |  |  |
| Strap muscles | 2.12 | 1.70-2.64 | ＜0.01 | 1.48 | 1.18-1.87 | ＜0.01 |
| T4a | 13.45 | 11.50-15.72 | ＜0.01 | 3.70 | 3.11-4.41 | ＜0.01 |
| T4b | 22.64 | 18.86-27.16 | ＜0.01 | 4.89 | 3.99-5.99 | ＜0.01 |
| 1-4cm | 2.45 | 2.02-2.97 | ＜0.01 | 1.62 | 1.33-1.98 | ＜0.01 |
| ＞4cm | 11.42 | 9.32-13.99 | ＜0.01 | 3.60 | 2.87-4.51 | ＜0.01 |
| N1a | 2.98 | 2.55-3.48 | ＜0.01 | 2.38 | 2.02-2.82 | ＜0.01 |
| N1b | 6.43 | 5.50-7.52 | ＜0.01 | 3.33 | 2.80-3.96 | ＜0.01 |
| M1 | 28.43 | 24.42-33.09 | ＜0.01 | 5.64 | 4.76-6.68 | ＜0.01 |

Definitions of the T, N and M components are based on the eighth edition TNM staging system.

HR, hazard ratio. CI, confidence interval.

eTable 2 The rules for the decision tree classification

| Rule | Variable |  |  |  |  |  |  |
| --- | --- | --- | --- | --- | --- | --- | --- |
|  | DM | ETE | Age | TS cm | LNM | n | CSS |
| 1 | No | no or only strap muscles | ＜55 | - | - | 34788 | 99.6% |
| 2 | No | no or only strap muscles | ≥55 | ≤4 | - | 14344 | 97.9% |
| 3 | No | no or only strap muscles | ≥55 | ＞4 | N0 | 668 | 95.7% |
| 4 | No | no or only strap muscles | ≥55 | ＞4 | N1 | 481 | 85.2% |
| 5 | No | T4 | ＜55 | - | - | 991 | 96.7% |
| 6 | No | T4 | ≥55 | ≤4 | - | 695 | 81.7% |
| 7 | No | T4 | ≥55 | ＞4 | - | 296 | 66.2% |
| 8 | Yes | - | ＜55 | - | - | 259 | 83.0% |
| 9 | Yes | no or only strap muscles | ≥55 | ≤4 | - | 174 | 75.3% |
| 10 | Yes | no or only strap muscles | ≥55 | ＞4 | - | 71 | 50.7% |
| 11 | Yes | T4 | ≥55 | - | - | 150 | 39.3% |

DM, distant metastasis. ETE, extrathyroidal extension.TS, tumor size. LNM, lymph node metastasis. CSS, cancer-specific survival.No, no or only strap muscles.
